# Utilization of fluid-based biomarkers as endpoints in disease-modifying clinical trials for Alzheimer’s disease: a systematic review

**DOI:** 10.1186/s13195-024-01456-1

**Published:** 2024-04-27

**Authors:** Marlies Oosthoek, Lisa Vermunt, Arno de Wilde, Bram Bongers, Daniel Antwi-Berko, Philip Scheltens, Pieter van Bokhoven, Everard G. B. Vijverberg, Charlotte E. Teunissen

**Affiliations:** 1 Department of Laboratory Medicine, Neurochemistry Laboratory, Vrije Universiteit Amsterdam, Amsterdam UMC, De Boelelaan 1117, 1081 HV Amsterdam, The Netherlands; 2 EQT Life Sciences, Johannes Vermeersplein 9, 1071 DV Amsterdam, The Netherlands; 3 Alzheimer Center, Department of Neurology, Vrije Universiteit Amsterdam, Amsterdam UMC, De Boelelaan 1117, 1081 HV Amsterdam, The Netherlands; 4 IXA - Amsterdam Neuroscience, Amsterdam UMC, Amsterdam, The Netherlands

**Keywords:** Alzheimer, Clinical trial, Fluid-based biomarker

## Abstract

**Background:**

Clinical trials in Alzheimer’s disease (AD) had high failure rates for several reasons, including the lack of biological endpoints. Fluid-based biomarkers may present a solution to measure biologically relevant endpoints. It is currently unclear to what extent fluid-based biomarkers are applied to support drug development.

**Methods:**

We systematically reviewed 272 trials (clinicaltrials.gov) with disease-modifying therapies starting between 01–01-2017 and 01–01-2024 and identified which CSF and/or blood-based biomarker endpoints were used per purpose and trial type.

**Results:**

We found that 44% (*N* = 121) of the trials employed fluid-based biomarker endpoints among which the CSF ATN biomarkers (Aβ (42/40), p/tTau) were used most frequently. In blood, inflammatory cytokines, NFL, and pTau were most frequently employed. Blood- and CSF-based biomarkers were used approximately equally. Target engagement biomarkers were used in 26% (*N* = 72) of the trials, mainly in drugs targeting inflammation and amyloid. Lack of target engagement markers is most prominent in synaptic plasticity/neuroprotection, neurotransmitter receptor, vasculature, epigenetic regulators, proteostasis and, gut-brain axis targeting drugs. Positive biomarker results did not always translate to cognitive effects, most commonly the small significant reductions in CSF tau isoforms that were seen following anti-Tau treatments. On the other hand, the positive anti-amyloid trials results on cognitive function were supported by clear effect in most fluid markers.

**Conclusions:**

As the field moves towards primary prevention, we expect an increase in the use of fluid-based biomarkers to determine disease modification. Use of blood-based biomarkers will rapidly increase, but CSF markers remain important to determine brain-specific treatment effects. With improving techniques, new biomarkers can be found to diversify the possibilities in measuring treatment effects and target engagement. It remains important to interpret biomarker results in the context of the trial and be aware of the performance of the biomarker. Diversifying biomarkers could aid in the development of surrogacy biomarkers for different drug targets.

**Supplementary Information:**

The online version contains supplementary material available at 10.1186/s13195-024-01456-1.

## Background

Clinical trials in Alzheimer’s disease (AD) historically have had high failure rates due to several reasons, one of which is the lack of tools to measure target engagement and other biologically relevant modulations to inform drug development [1]. Biomarkers may present a solution to measure biologically relevant endpoints, which are required to demonstrate treatment effects on the underlying pathology. Positron emission tomography (PET) is commonly used to detect a treatment response and closest to becoming a surrogate endpoint. Yet, PET measurements are still largely limited to amyloid or tau total accumulation status, while fluid-based biomarkers offer the advantage of measuring several biological processes simultaneously in the same sample. This is directly relevant for non-amyloid and non-tau targets but also to detect a pattern of downstream biological changes. In addition, fluid markers offer the possibility of repeated sampling without radiation risk, and lower costs compared to PET. Lastly, fluid-based biomarkers can form a faster dynamic alternative to PET scans.

Biomarker endpoints can be used as primary, secondary or exploratory endpoints and can be divided into those demonstrating treatment effects on pathological processes or ‘target engagement biomarkers’. Target engagement markers should be related to the modulation of the molecular target of the investigational product and are therefore highly specific to the drug and target class. Fluid-based biomarkers can also be used as surrogate markers when they predict a future clinical benefit [2]. Generally, the use of biomarkers highly depends on the specific context, such as the patient population, duration of the trial, and the investigational product. Depending on the use, an important consideration is confidence in the performance of the biomarker. For primary endpoints a biomarker is ideally well-established in terms of disease relationship and assay qualities. However, due to the specific nature of target engagement markers, information regarding these markers can be limited in relation to the disease and technically advanced assays may not always be readily available.

Changes in biomarkers demonstrating treatment effect should be representative of altered underlying pathology. Examples of fluid-based biomarkers measurable in cerebrospinal fluid (CSF) and blood potentially fit for this purpose are phosphorylated Tau (pTau) isoforms [3–5], amyloid β (Aβ) peptides [6–11], neurofilament light (NFL) [12–14], indicative of axonal damage, and glial acidic fibrillary protein (GFAP) [15], related to astrogliosis. PTau isoforms, NFL and GFAP change with disease progression and have monitoring ability across the AD continuum, and are therefore of particular relevance in AD trials [16–25]. This is further supported by the significant effect of lecanemab [26] and donanemab [27] on pTau181, pTau217 and GFAP. Furthermore, fluid-based biomarkers can also indicate target engagement as shown by the increase in CSF Aβ42 and plasma Aβ42/40 in the lecanemab trial [26].

Within the oncology and cardiology field, biomarker-supported drug development is widely implemented and supported by regulatory agencies to obtain market approval and reduce trial failure rates [28]. Examples include carcinoembryonic antigen or prostate-specific antigen for oncological drugs and low-density lipoprotein cholesterol and blood pressure for cardiovascular drugs [29, 30].

To determine the gaps and opportunities of fluid-based biomarker endpoints in AD clinical trials, we conducted a systematic review of which and how frequently fluid-based biomarkers have so far been employed for what purpose (primary, secondary or exploratory endpoint or target engagement) and in which type of clinical trials (phase, patient population, drug target class). By doing so, we can put trends, gaps, and opportunities in biomarker development to support outcomes of AD clinical trials in context.

## Methods

### Selection of trials

To identify how fluid-based biomarkers are used in clinical trials, we performed a search using the search term ‘Alzheimer’s Disease’ on www.clinicaltrials.gov (8-FEB-2022). A selection was made for interventional studies that had a study start date between January 1st 2017 and January 1st 2022. We excluded trials that had an unknown status when the search was performed. We only included medicinal products suspected to be disease-modifying therapies (DMTs; i.e. treatments that slow or stop AD progression by targeting the underlying pathology), and which were either in phases 1, 2, 3 or 4. Trials included healthy volunteers, asymptomatic at-risk participants, patients with mild cognitive impairment (MCI) due to AD, AD dementia or a combination. There were no requirements on sample sizes or study duration. Open-label extension studies and sub-studies were accommodated with their respective original trial to avoid double scoring.

Excluded trials were grouped into the following categories. 1) Non-AD trials, which included trials where the goal was not related to treating AD patients, but for instance, PET tracer interrogation, interventions targeting caregivers, or trials focusing on neurodegenerative diseases in general. 2) Drugs focusing on psychiatric alleviation or other symptom-reducing medication, among which trials investigating cholinesterase inhibitors or N-methyl-D-aspartate receptor antagonists. 3) Trials of non-pharmacologic therapeutic approaches such as cognitive training and health tech interventions.

### Categorization of the trials

For each trial, all information provided on clinicaltrials.gov was tabulated, listing the NCT number, trial phase, treatment period in weeks, trial geography, inclusion diagnosis, and sponsor/collaborator. Trials with a double phase classification, i.e., Phase 1|Phase 2 or Phase 2|Phase 3 were classified as the most advanced phase (phase 2 and phase 3, respectively) throughout the analysis. Throughout the analysis, trials registered in phases 3 and 4 were combined. In the text we will refer to trials in phase 3 and 4 as trials in phase 3. Since there were only six trials in phase 4, this was not informative as a separate category. When a trial took place on three or more continents, it was classified as global. To visualize the use of fluid-based biomarkers to the type of sponsor, we classified the sponsor as big pharma, biotech, academia or a combination of industry (including both pharma and biotech) and academia.

### Categorization of targets

The scoring of the target class was classified based on the Common Alzheimer’s Disease Research Ontology (CADRO) developed by the National Institute on Aging and the Alzheimer’s Association using category C 3 classes [31]. In line with our previous publication [32] we added endoplasmic reticulum (ER) stress/cellular stress, lysosomal, endosomal, autophagy, and antiviral/antibacterial as extra target classes.

### Categorization of fluid-based biomarker endpoints

Endpoints were divided into primary, secondary and exploratory endpoints following clinicaltrials.gov. If one endpoint was used in several ways (primary, secondary or exploratory) only the most important endpoint was indicated (in order from most to least important: primary, secondary, and exploratory). The fluid-based biomarker endpoints were further specified as CSF or blood-based biomarkers. We summarized which fluid-based biomarkers were used as what type of endpoint. We combined NFL, GFAP, YKL-40, inflammatory cytokines, and other less commonly used biomarkers into one group for the analysis: other. Combining them allowed for a better general overview. We also indicated if and how target engagement markers were used and if so which one.

After finalizing the scoring process, a Pubmed search based on NCT number was performed to determine if published results were available for finalized trials.

## Results

### Trial characteristics

**Fig. 1:**
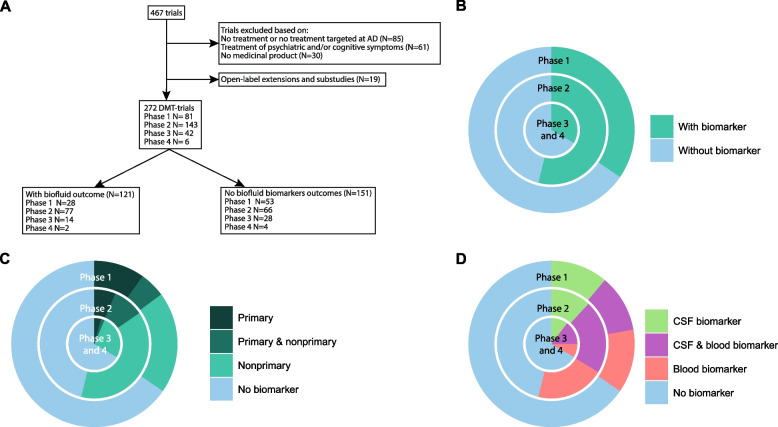
Trial selection and use of fluid-based biomarker endpoints in clinical trials investigating Alzheimer disease-modifying treatments per development phase. Overview of the methods (**A**). Percentage of trials using a fluid-based biomarker (**B**). Percentage of trials using a fluid-based biomarkers as primary, primary and nonprimary, or nonprimary endpoint (**C**). Percentage of trials using CSF, blood or both (**D**)

Of the 467 search results, 272 were included based on eligibility (Fig. 1A). Of these trials, 30% (*N* = 81) were in phase 1, 53% (*N* = 143) in phase 2, and 18% (*N* = 48) in phase 3. Most trials took place in North America (*N* = 140), Europe (*N* = 40) or globally (*N* = 28) (Supplementary Table 1). In terms of sponsors, 47% (*N* = 128) was sponsored by biotech, 24% (N = 66) by academia, 20% (N = 55) by big pharma, and 8% (*N* = 23) by a combination of academia and industry (Supplementary Fig. 1).

### Characteristics of trials with and without fluid-based biomarker endpoints

Of the 272 trials, 44% (*N* = 121) used fluid-based biomarker endpoints (Fig. 1A) and 56% (*N* = 151) did not. Of the trials with fluid-based biomarker endpoints, 23% (*N* = 28) were in phase 1, 64% (*N* = 77) in phase 2, 13% (*N* = 16) in phase 3. On average, 157 participants were included (IQR: 20–185), who were treated for an average of 46 weeks (IQR: 12–52). AD dementia (18%; *N* = 49, Table 1) was the most frequent trial population, followed by a combination of patients ranging from MCI due to AD to AD dementia (15%; *N* = 40). Biomarker use was most prominent in academia-sponsored trials, 71% (*N* = 47) of trials sponsored by academia employed fluid-based biomarkers. Although biotech sponsored most trials, only 36% (*N* = 46) of the trials sponsored by biotech used fluid-based biomarkers (Supplementary Fig. 1).

**Table 1:** Fluid-based biomarker endpoint use per diagnostic group and trial phase. Percentage in N trials without (w/o) and with (w/) fluid-based biomarker endpoints of all 2,72 trials and percentage in N trials with fluid-based endpoints per phase. Due to rounding the percentages may not add up to 100%

Diagnostic group	N Trials w/o fluid-based endpoint	N Trials w/ fluid-based endpoint	N Trials w fluid-based endpoint		
			**Phase 1**	**Phase 2**	**Phase 3 + 4**
Healthy participants	32 (12%)	7 (3%)	6 (5%)	1 (0.8%)	–
Asymptomatic at risk	4 (1%)	9 (3%)	1 (0.8%)	4 (3%)	4 (3%)
Asymptomatic at risk to MCI due to AD	1 (0.4%)	0 (0%)	–	–	–
Asymptomatic at risk to AD dementia	1 (0.4%)	4 (1%)	1 (0.8%)	3 (2%)	–
MCI due to AD	8 (3%)	12 (4%)	3 (2%)	6 (5%)	3 (2%)
MCI due to AD to AD dementia	42 (15%)	40 (15%)	11 (9%)	25 (21%)	4 (3%)
AD dementia	63 (23%)	49 (18%)	6 (5%)	38 (31%)	5 (4%)

Of the trials that did not employ fluid-based biomarker endpoints, 35% (*N* = 53) were in phase 1, 44% (*N* = 66) were in phase 2, and 21% (*N* = 32) were in phase 3. These trials included 267 participants on average (IQR: 36–233) and participants were treated for an average of 35 weeks (IQR: 4–52). In trials that did not use fluid-based endpoints, 23% (*N* = 63) of the trials included AD dementia patients (Table 1), also followed by MCI due to AD to AD dementia (15%; *N* = 42).

### Fluid-based biomarker endpoints by phase

In each of the phases of drug development, blood and CSF biomarkers serve a different function. In our review, we found that fluid-based biomarker endpoints were most prominent in phase 2 trials (54%, *N* = 77; Fig. 1B). In phases 1 and 3 a little over 1/3 of the trials used fluid-based endpoints. The lowest utilization of fluid-based biomarkers as primary endpoint was in phase 3 (15% (*N* = 12) of the phase 1, 15% (*N* = 22) of the phase 2, and 6% (*N* = 3) of the phase 3 trials; Fig. 1C). The use of blood and CSF markers was roughly similar throughout the development phases. Approximately 1/3 used CSF, 1/3 used blood, and 1/3 used a combination of both CSF and blood (Fig. 1D).

### Fluid-based biomarker endpoints per type of biomarker and by stage

In Table 2 we provide an overview of which markers are used for what purpose. From the three main categories, Aβ, Tau and other, Tau was used most frequently in CSF and other biomarkers in blood. As secondary endpoints, Aβ and Tau in CSF were the main biomarkers used. In blood, two major markers used as secondary endpoint were the inflammatory cytokines (*N* = 16) and NFL (*N* = 13). Only 10 trials employed GFAP in blood as an endpoint.

**Table 2:** Number of trials using a specific marker as primary, secondary, or exploratory endpoint per matrix. Unspecified indicates the trial mentioned using Tau or Aβ but not which isoform or species. Rest biomarkers include the biomarkers that are target specific and used less frequently; in CSF: fatty acid levels, microtubule binding region Tau, neurogranin (NRGN), sTREM2, HMGB1, Aβ oligomers, calcineurin, reverse transcriptase activity, apolipoproteins and HDL concentration; in blood: glucose, fatty acid, and ketone levels, receptor mediators of ketone metabolism in plasma exosomes autophagy markers, plasma exosomes, COX/CS activity, 24-hydroxycholesterol, hormone levels, anti-P. gingivalis IgG, apolipoproteins and HDL concentration, Aβ oligomers, NRGN, calcineurin, BACE1 concentration, monocyte CD16 and HLA-DR expression, reverse-transcriptase activity, SASP, CD3, p16INK4A + as senescence markers, SavaDx, eotaxin-1, TSPO phenotype, PRA, sTREM2, gut microbiome, and extracellular vesicles concentrations

CSF	Primary (26/121)	Secondary (52/121)	Exploratory (14/121)	Blood	Primary (21/121)	Secondary (55/121)	Exploratory (20/121)
**Aβ (at least one of the below)**	**6**	**36**	**14**	**Aβ (at least one of the below)**	**4**	**25**	**19**
Aβ40	3	17	4	Aβ40	4	13	6
Aβ42	4	23	5	Aβ42	4	13	6
Aβ42/40 ratio	2	11	6	Aβ42/40 ratio	4	7	7
Unspecified Aβ	1	13	6	Unspecified Aβ	0	11	6
**Tau (at least one of the below)**	**11**	**47**	**10**	**Tau (at least one of the below)**	**3**	**26**	**12**
pTau isoforms	10	32	4	pTau isoforms	3	17	6
tTau	5	32	5	tTau	1	8	4
Unspecified Tau	0	11	5	Unspecified Tau	0	8	5
**Other (at least one of the below)**	**18**	**26**	**6**	**Other (at least one of the below)**	**18**	**46**	**12**
GFAP	0	2	2	GFAP	0	8	2
NFL	5	13	4	NFL	3	15	4
Inflammatory cytokines	5	5	1	Inflammatory cytokines	2	18	7
YKL-40	3	4	4	YKL-40	0	0	0
Rest	14	17	5	Rest	14	30	10

**Fig. 2:**
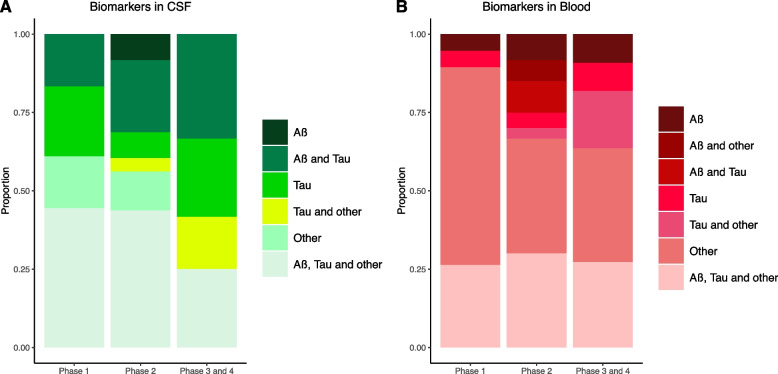
Proportion of biomarker or biomarker combinations used in CSF or blood. Trials including CSF biomarkers as endpoints (**A**) per phase (phase 1: *N* = 18; phase 2: *N* = 48; phase 3 and 4: *N* = 12). Trials including blood biomarkers as endpoints (**B**) per phase (phase 1: *N* = 19; phase 2: *N* = 60; phase 3 and 4: *N* = 11). Other biomarkers included GFAP, NFL, YKL-40, inflammatory cytokines, and mechanism specific biomarkers

In general, the majority of the trials that use CSF markers, included Tau-related biomarkers (Fig. 2A) and often in combination with Aβ or other biomarkers. In blood, there is an increase in the use of Tau-related biomarkers in phase 2 and 3 (Fig. 2B). Biomarkers from the “other” category were most popular in blood. In phase 1 these were used primarily independently, and in the later phases more often in combination with AD pathology markers.

### Fluid-based target engagement markers per phase

**Fig. 3:**
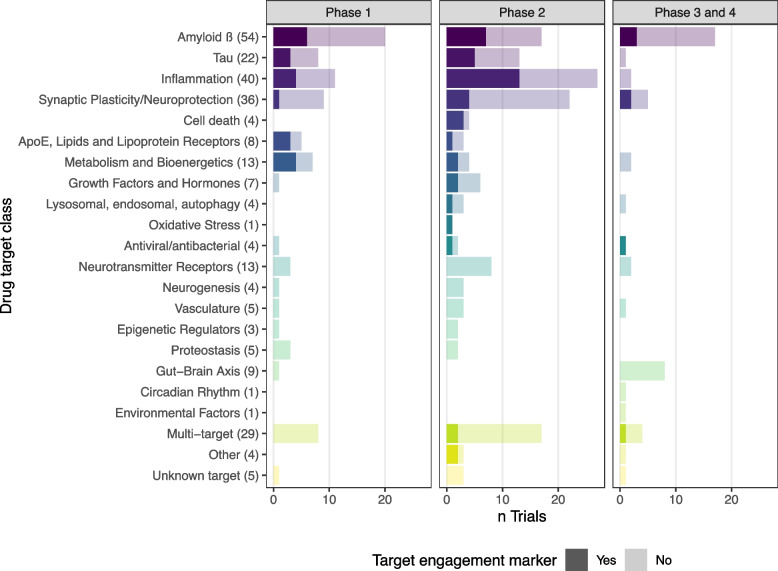
Number of trials with and without a fluid-based target engagement marker, by target class. Total number of trials (*N* = 272) was included in this analysis, darker shade indicates use of a fluid-based target engagement marker. Between brackets the total number of trials in that target class is listed

Fluid-based target engagement markers were employed in 26% (N = 72) of the trials (26% of the phase 1 trials, 31% of the phase 2 trials, 15% of the phase 3 trials; Fig. 3). Drugs targeting inflammation (17 of 40) and amyloid (16 of 54) used fluid-based target engagement markers most often. Lack of target engagement marker use was most apparent in drugs targeting synaptic plasticity/neuroprotection (7 of 36 trials), despite the availability of markers. Several target classes did not include any fluid-based target engagement marker, namely neurotransmitter receptors, neurogenesis, vasculature, epigenetic regulators, proteostasis and, gut-brain axis, environmental factors, multi-target, and unknown targets. Supplementary Table 2 provides a list of all target engagement markers used per target class.

### Fluid-based biomarker results in relation to cognitive results

Twenty trials had published the results (Supp. Table 3, Table 3). Seven of these trials (Gantenerumab, Neflamapimod, 3TC, MAPTrx, Gosuranemab, Semorinemab, and Zagotenemab) showed significant effects on at least one fluid-based biomarker, but no effect on the clinical endpoints and three trials (Donanemab [2x] and Lecanemab) were positive on both bliofluid markers and cognitive endpoints. None of the trials reported only positive effects on cognition. The biomarker most commonly affected by the treatment were pTau isoforms.

**Table 3:** Overview fluid-based endpoint results in trials

The TRAILBLAZER-ALZ study, investigating donanemab (target class: amyloid β), showed no significant changes in plasma Aβ42/40 ratio levels [27]. There was a significant decrease in plasma pTau217 and GFAP reported in patients receiving donanemab. These changes also correlated with change in brain amyloid plaques as visualized by PET. Donanemab slowed cognitive decline compared to placebo.
Lecanemab (target class: amyloid β) showed a significant increase in CSF Aβ42 after 12 and 18 months in people receiving the drug [26]. There was no change in the Aβ40 measurements between placebo and treatment. Furthermore, levels of CSF tTau, pTau181, and NRGN were reduced after 12 and 18 months. No change in CSF NFL was reported between the two groups. In plasma, Aβ42/40 ratio increased reported and plasma pTau181 and GFAP decreased following lecanemab treatment compared to placebo. Patients receiving lecanemab showed reduced rates in cognitive decline compared to placebo.
Gantenerumab and crenezumab (target class: amyloid β) both showed insignificant treatment effects. There was no significant effect on cognition for patients receiving gantenerumab [33]. CSF pTau181, tTau, and NRGN decreased in the patients receiving gantenerumab. However, different responses in fluid biomarkers were found for men and women [34]. Crenezumab did not show changes in the core AD biomarkers measured: CSF Aβ42, Aβ40, tTau, and pTau181 [35].
Semorinemab, gosuranemab, and tilavonemab (target class: Tau) are all monoclonal antibodies investigated in phase 2 trials which showed no clinical benefit. Semorinemab showed dose-dependent increase in plasma mid-domain tau, which is indicated as their target engagement marker and a lowering in CSF pTau181, pTau217 and tTau [36, 37]. Gosuranemab also showed target engagement by lower CSF N-terminal tau. However, there was no effect on Tau PET [38]. Tilavonemab reduced CSF free Tau in a dose-dependent manner after 12 weeks and increased plasma tTau, also indicating target engagement [39]. MAPT_rx_ is an antisense oligonucleotide (target class: Tau) that has shown a dose-dependent effect on CSF tTau concentrations [40].
Neflamapimod (target class: inflammation), a p38α kinase inhibitor, showed reduced CSF levels of pTau181 and tTau compared to placebo and a positive trend for NRGN. No significant effects were seen for NFL, Aβ42, and Aβ40 levels [41]. There was no effect reported on episodic memory performance (HVLT-R).
A trial with rifaximin (target class: antiviral/anti-bacterial), which is an antibiotic that reduces neurotoxic microbial drivers of inflammation by changing gut flora composition, also measured several fluid-based markers. However, there were no significant changes in the BTE inflammatory cytokines. NFL was significantly lower following treatment [42].
S-equol (target class: growth factors and hormones), an estrogen receptor β agonist inducing mitochondrial activity, used cytochrome oxidase (COX) and citrate synthase (CS) activity in platelet-derived mitochondria as target engagement marker. COX/CS activity increased for 11/15 patients following two weeks of study drug administration [43].

## Discussion

We provide a comprehensive overview of the use of fluid-based biomarkers in AD trials starting between 01-01-2017 and 01-01-2024, evaluating the frequency and purpose of utilization of these biomarkers as endpoint. Overall, 44% of the trials used fluid-based biomarkers as an endpoint to monitor either biological treatment effects and/or target engagement. Biomarkers to show biological treatment effects were employed as a primary endpoint most often in phase 2, and the percentage of fluid-based biomarkers as primary endpoint decreased in phase 3 trials. This was expected given that the purpose of phase 3 trials is to show a clinical benefit. CSF and blood-based endpoints were used approximately equally, which was unexpected considering the burden of repeated CSF sampling. The classical pathologic AD ATN markers, Aβ, pTau isoforms and tTau, were used most often in CSF and not yet in blood. Use of target engagement markers was limited (26%). Furthermore, there were several drug target classes that did not include any fluid-based target engagement markers, including drugs targeting neurotransmitter receptors, neurogenesis, vasculature, epigenetic regulators, proteostasis, and the gut-brain axis. We also show there are several trials that have reported biomarker findings, without positive clinical findings. Combined, these findings show that there is an unused potential for the use of fluid-based biomarkers and a need for novel fluid biomarkers to fully capture the complex biology of the disease and for further implementation in clinical trials.

### Treatment effect biomarkers are needed in disease-modifying trials

**Fig. 4:**
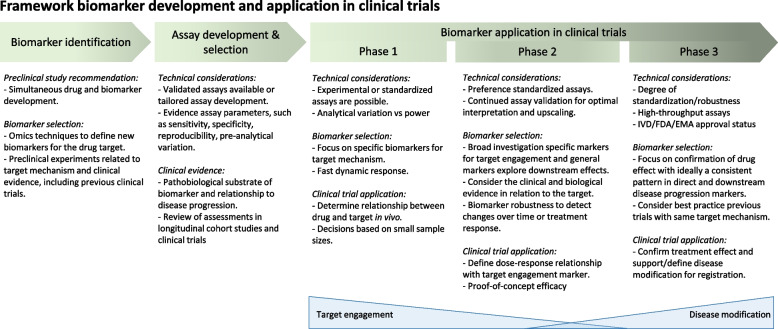
Framework biomarker development and application in clinical trials

We show that 44% of the trials included in this study used fluid-based biomarker endpoints, mostly in phase 2 (54%). Throughout all phases, fluid-based biomarkers were mostly employed as exploratory endpoints. However, as the goals of the field shift to primary prevention, inclusion of biomarkers as primary endpoints is crucial, as determining efficacy based purely on cognitive endpoints will become more challenging [44, 45]. Specific considerations on biomarker application in clinical trials are shown in Fig. 4. Ideal fluid biomarkers have been investigated in the context of both the disease, the drug mechanism, and are technically mature.

The classical AD biomarkers [46] were employed frequently and can reliably be measured in CSF and blood [6, 17, 47–50]. Changes in these biomarkers could be indicative of treatment effects and disease modification. Additionally, we show that GFAP was used in only 10 trials. Recent studies show that especially plasma GFAP rather than CSF GFAP correlates with Aβ pathology and has a high prognostic value [22–24, 51]. Therefore, GFAP might be additive to Aβ and pTau, which are often directly modified by the drugs, as a marker of disease modification in the early stages of AD. While treatment effect on GFAP might not be required for FDA approval, it can be viewed as strong evidence for a downstream effect of disease modification because it is not related to the target. A decrease in GFAP could be indicative of slowing disease progression, but longer follow-up is needed to prove this hypothesis and better understand the mechanism [23]. The donanemab and lecanemab trials showed its responsiveness in patients with early symptomatic AD [26, 27].

### Future of both CSF and blood-based biomarkers

One-third of the trials that employed fluid-based biomarker endpoints used a combination of both CSF and blood-based biomarkers, another third only used CSF biomarkers and the last third used only blood-based biomarkers. CSF and blood-based biomarkers both have their own advantages. The advantage of using CSF is its close proximity to the brain, thereby likely providing a reflection of ongoing brain pathology without peripheral effects on the biomarker levels [52]. Blood-based biomarkers are susceptible to metabolism and excretion interference but offer a low invasive alternative to CSF [53]. This obvious advantage allows for easier serial analyses which can thereby promote trial participation.

### Fluid-based target engagement markers are not used to the fullest potential

Fluid-based target engagement can be useful to demonstrate preclinical to clinical translation in early drug development (Fig. 4). Proving target engagement as early as possible saves time and resources, for example by enabling a Bayesian trial design [54], risk of failure of later-stage trials can potentially be reduced [55]. When target engagement is demonstrated, but no effect on cognition is found, this could indicate that the target is not fit or a different approach is needed. This is demonstrated by the results of Semorinemab, gosuranemab, and tilavonemab (Supp. Table 3; Table 3) [36, 38, 39]. The biomarker findings indicate there is target engagement, however, this is not translated to disease modification and clinical benefit. These drugs have not been further investigated in larger trials.

Only 7 of the 36 trials that investigated drugs targeting synaptic plasticity/neuroprotection employed target engagement markers, even though multiple markers are available. NRGN, a post-synaptic marker was used most frequently, i.e. 5 times. CSF presynaptic synaptosomal-associated protein 25, vesicle-associated membrane protein-2, and growth-associated protein 43 (GAP43) have recently been described as synaptic biomarkers, and can likely provide information on presynaptic integrity [56–59]. β-synuclein, which can be measured in both CSF and blood and relates to Aβ-pathology, could also be a useful marker to investigate synaptic integrity [60–62]. Due to limited data from trials, it is unknown which of the synaptic markers, or a panel could detect treatment effects on synaptic function. Therefore, it is too early to conclude whether pre- or post-synaptic markers are more appropriate, or have added value for showing target engagement. By including them in trials at the early stages we also generate insights into the performance of the biomarkers and which assays are suited for clinical trial interpretation. Using both pre- and postsynaptic markers in combination can generate insight into the synaptic health of the neurons and target engagement.

Strikingly, there are several target classes not using any fluid-based marker, even though there are markers available for some of these. These include drugs targeting neurotransmitter receptors, neurogenesis, vasculature, epigenetic regulators, proteostasis, and the gut-brain axis. Potential markers for medication focusing on vasculature include vascular cell adhesion molecule-1 (VCAM-1) or intercellular adhesion molecule-1 (ICAM-1), markers related to vascular endothelium [63, 64]. Research indicated that higher levels of VCAM-1 and ICAM-1 were associated with increased Aβ and tau pathology [65]. Soluble platelet-derived growth factor receptor-β, a pericyte marker, or vascular endothelial-cadherin (VEC), a marker for endothelial injury can give information on blood–brain-barrier integrity [66, 67]. VEC concentrations are increased in preclinical AD [67] and this marker could therefore be implemented in trials focusing on early AD stagesfor potential use as both a target engagement marker or to demonstrate disease modification even in these early stages.

### The importance of regulatory status for biomarker implementation in clinical trials

The guidance documents of the US Food and Drug Administration (FDA) and the European Medicines Agency (EMA) focus on cognitive effects to determine efficacy in AD clinical trials. However, both also indicate that biomarkers should be used to support disease modification [68, 69]. Recently, the FDA has shown to be increasingly open to the use of biomarkers as surrogate endpoints in neurodegenerative diseases. The agency gave Accelerated Approval of aducanumab and lecanemab for AD, and tofersen for amyotrophic lateral sclerosis (ALS), indicating that these treatments demonstrated ‘an effect on a surrogate endpoint (Aβ PET for AD and CSF NFL for ALS) that is reasonably likely to predict a clinical benefit to patients’ [70–73]. While the use of fluid biomarkers for AD trial population enrichment has official support from these agencies, such approvals are not available for biofluid endpoints [74, 75]. In a recent support letter, the EMA recommended the monitoring ability, intra-individual variability, population variability, and behavior over time of the biomarkers needs to be established for such qualification approval [76]. The qualification approval of fluid-based biomarkers as clinical trial endpoints can advance their role in treatment evaluation. Moreover, approval by regulatory agencies could provide an incentive for big pharma to implement them in larger trials also as primary endpoint. Here we show only 27% of trials sponsored by big pharma employed fluid biomarkers. With more big pharma implementing biomarkers, the field can gain insights into their specific uses within a trial setting and more data on the biomarkers over time will be generated, especially if trial data is published and shared. This aid in the interpretation of biomarker results in relation to clinical endpoints to establish insights into the effect sizes required for clinical benefit.

### Future perspectives

With current technological advances, we are able to quickly analyze a significant amount of proteins with higher accuracy to establish new potential markers. A recent study on the CSF proteome identified new non-amyloid-related endpoint markers [77]. This could aid with the development of biomarkers for target classes where there is no or few biomarkers available and offer new ways to measure general biological effects. With improved technologies the biomarkers can be combined and multiplexed, which allows for a large number of proteins to be measured quickly making trial analyses easier [53, 78, 79]. Furthermore, advances in technological sensitivity will aid the development of novel blood-based biomarkers.

The aducanumab, donanemab, lecanemab and gantenerumab trials (Table 3) can give insights into the relation of several biomarkers with cognitive outcomes. This can bring us closer to the holy grail of surrogate biomarkers. Based on the study findings, CSF pTau181 and plasma pTau217, pTau181, and GFAP seem to have the most potential for surrogacy biomarkers in the amyloid pathway. Surprisingly, NFL did not respond to treatment in those trials, while in multiple sclerosis (MS) trials this is a very good marker for treatment monitoring, and evidence is developing for ALS [80–82]. Potentially, NFL effects are further downstream in AD compared to MS and ALS, thus effects take longer to be visualized in AD. Moreover, there is a bigger relative increase compared to healthy age-matched controls in MS and ALS compared to AD [83]. In order to substantiate the use of fluid-based biomarkers as surrogate endpoints in AD trials, understanding the relation of biomarker dynamics, e.g. if biomarker reduction below a certain threshold, within a critical time-window or between different groups (e.g. sex, APOE4 carriers) is required, will be key in the implementation as surrogate endpoints.

### Limitations of the study

Not all biomarker analysis plans may be registered on clinicaltrials.gov. Often there is a significant time period between trial initiation and the end of trial date. Development of biomarkers may have significantly changed nearing the end of trial, and analysis plans may be finalized towards the trial completion, while novel biomarkers can also be included in post-hoc analysis. This might lead to underrepresentation of certain biomarkers in this analysis.

A downside of the biofluid biomarker field is the variable level of validation of the assays, ranging from very standardized high throughput to explorative assays with high CV. Therefore the power of the studies, and risk of false positive and false negative findings is difficult to estimate.

A lot of the studies included in this analysis are not yet finished, so it cannot be investigated if trials with biomarkers have higher success rates. As more successful trials are needed to definitely determine the future role of certain biomarkers, it would be interesting to see the final results of the biomarkers in relation to clinical outcomes. Several trials reported changes in biomarkers and some of these together with clinical effects. Whether biomarker thresholds for clinical benefit can be established, becomes a testable hypothesis as more data is becoming available. To facilitate this, the assay standardization efforts are very important because it enhances the comparability. In addition, a structured re-analyses, engaging the trial investigators, using meta-analyses techniques that account for assay, design, population and trial mechanisms differences could be used to estimate these response relationship and possibly thresholds in a similar manner as has been done for amyloid PET [84].

## Conclusion

In conclusion, fluid biomarkers offer a way of measuring biological endpoints and a range of markers are used commonly within the AD clinical trial setting. For the near future, there will be a rapid uptake of the low-invasive blood biomarkers, but we foresee CSF markers will remain important to determine brain-specific treatment effects on an expanding range of disease-modifying mechanisms tested in the interventions. We also identified that there still exists a need for new fluid biomarkers, to monitor biological effects and target engagement. Recently developed biomarker detection technologies offer a solution to finding such markers. Qualification approval of fluid-based biomarkers is needed to advance their use as endpoints in AD clinical trials, in parallel to solving the outstanding questions regarding which markers are suitable to prove surrogacy. These gaps would have to be addressed before biomarker implementation in clinical trials is as advanced as seen in the oncology or cardiology fields. With increased activities towards new drug development, disease-modifying treatments and more successful trial data that will become available, the prospect of overcoming these gaps also draws closer.

### Supplementary Information

**Additional file 1. **Overview of sponsor types and fluid-based biomarker use per development phase.

**Additional file 2. **

**Additional file 3. **

**Additional file 4. **

## Data Availability

The datasets used and/or analysed during the current study are available from the corresponding author on reasonable request.

## Abbreviations

AD: Alzheimer’s disease
ALS: Amyotrophic lateral sclerosis
Aβ: Amyloid β
CADRO: Common Alzheimer’s Disease Research Ontology
COX: Cytochrome oxidase
CS: Citrate synthase
CSF: Cerebrospinal fluid
DMT: Disease-modifying thereapy
EMA: European Medicines Agency
ER: Endoplasmic reticulum
FDA: Food and Drug Administration
GAP43: Growth-associated protein 43
GFAP: Glial fibrillary acidic protein
ICAM-1: Intracellular adhesion molecule-1
IQR: Inter quartile range
MCI: Mild cognitive impairment
MS: Multiple sclerosis
NFL: Neurofilament light
NRGN: Neurogranin
PET: Positron emission tomography
pTau: Phosphorylated Tau
tTau: Total Tau
VCAM-1: Vascular cell adhesion molecule-1
VEC: Vascular endothelial cadherin
