# Supplementary figures and images for: Utilization of fluid-based biomarkers as endpoints in disease-modifying clinical trials for Alzheimer’s disease: a systematic review
Source: Alzheimers Res Ther. 2024 Apr 27;16:93. doi: 10.1186/s13195-024-01456-1 (PMC11055304; doi:10.1186/s13195-024-01456-1)

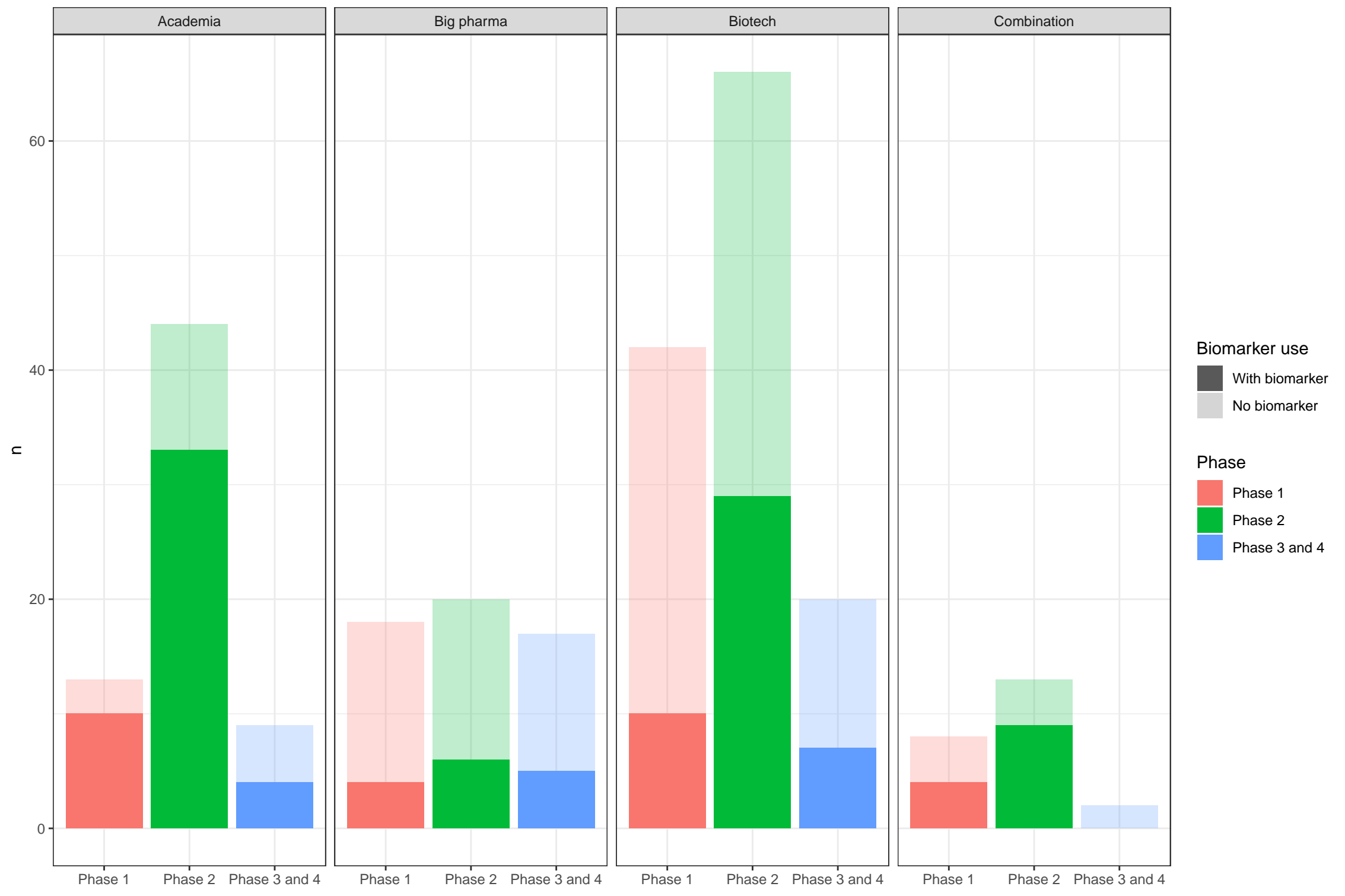

Supplement: Supplementary file 1 — Additional file 1. Overview of sponsor types and fluid-based biomarker use per development phase. [file 13195_2024_1456_MOESM1_ESM.pdf]
