# Supplementary material for: Utilization of fluid-based biomarkers as endpoints in disease-modifying clinical trials for Alzheimer’s disease: a systematic review
Source: Alzheimers Res Ther. 2024 Apr 27;16:93. doi: 10.1186/s13195-024-01456-1 (PMC11055304; doi:10.1186/s13195-024-01456-1)
Supplement: Supplementary file 2 — Additional file 2. [file 13195_2024_1456_MOESM2_ESM.docx]

**Supp. Table 1. Overview of participating countries in all trials.** Due to rounding the percentages do not add up to 100%

| Continent(s) | N trials (%) |
| --- | --- |
| North-America | 140 (53%) |
| Europe | 40 (15%) |
| Asia | 21 (8%) |
| Oceania | 12 (5%) |
| North-America & Europe | 13 (5%) |
| North-America & Oceania | 5 (2%) |
| North-America & Asia | 3 (1%) |
| Europe & Oceania | 1 (0.4%) |
| Global | 28 (11%) |
