# Supplementary material for: Utilization of fluid-based biomarkers as endpoints in disease-modifying clinical trials for Alzheimer’s disease: a systematic review
Source: Alzheimers Res Ther. 2024 Apr 27;16:93. doi: 10.1186/s13195-024-01456-1 (PMC11055304; doi:10.1186/s13195-024-01456-1)
Supplement: Supplementary file 3 — Additional file 3. [file 13195_2024_1456_MOESM3_ESM.docx]

**Supplementary table 2** **Overview of the use of fluid-based target engagement biomarkers per target class with indication of specific markers.** In case markers were used more often, they are only mentioned once.

| Target class | Target engagement fluid-based markers used |
| --- | --- |
| Amyloid β | Anti-Aβ40 antibodies; Aβ40 and Aβ42; anti-amyloid antibody titre; Aβ42/Aβ40 ratio; Aβ oligomers, sAPPa, sAPPb |
| Tau | Anti-tau antibody titres; MTBR-tau; tTau, free Tau and pTau, tau oligomers |
| Inflammation | GFAP, IFNg TNFa, TGFb1, CD33, sTREM2; immune risk profile CD4:CD8 ratio; IL-34 and CSF-1 levels; IL-1b, IL-2, IL-4, IL-5, IL-6, IL-8, IL-10, IL-12p70, IL-22; monocyte expression levels of CD16 and HLA-DR; Sema4D; calcineurin, MCP-1, CCL2 |
| Synaptic plasticity/ neuroprotection | NRGN; Aβ oligomers; linkage of FLNA to a7nAChR and TLR-4, synaptotagmin |
| Cell death | Senescence-Associated Secretory Phenotype composite score, Cluster of Differentiation 3, cyclin-dependent kinase inhibitor 2A, and T cells; IL-6 and P16 |
| ApoE, lipids, and lipoprotein receptors | Change in APOE2-APOE4 isoforms; 24-hydroxycholesterol; apolipoproteins and HDL concentration |
| Metabolism and bioenergetics | Total ketones; ketone production; glucose and insulin AUC; fatty acids; glucagon, receptors and mediators of ketone metabolism in plasma exosomes; |
| Growth factors and hormones | Testosterone, androstenedione, dehydroepiandrosterone sulfate, adrenocorticotropic hormone; COX/CS activity |
| Antiviral/antibacterial | Ammonia and cytokines; anti-P. gingivalis IgG |
| Lysosomal, endosomal, autophagy | Autophagy markers (not described); |
| Oxidative stress | 8-OHdG/8-OHG |
| Neurogenesis | None |
| Neurotransmitter receptors | None |
| Vasculature | None |
| Epigenetic regulators | None |
| Proteostasis | None |
| Gut-Brain axis | None |
| Environmental factors | None |
| Multi-target | GDK-3β activity, BDNF |
| Other | Reverse transcriptase activity; Excretion of Aβ40 and Aβ42 from the brain |
| Unknown target | None |
