# Supplementary material for: Utilization of fluid-based biomarkers as endpoints in disease-modifying clinical trials for Alzheimer’s disease: a systematic review
Source: Alzheimers Res Ther. 2024 Apr 27;16:93. doi: 10.1186/s13195-024-01456-1 (PMC11055304; doi:10.1186/s13195-024-01456-1)
Supplement: Supplementary file 4 — Additional file 4. [file 13195_2024_1456_MOESM4_ESM.docx]

**Supp. Table 3 Overview of available results.** Results for fluid-based biomarkers and clinical endpoints are summarized following a pubmed search with the NCT number. Information on inclusion criteria is reported as age, diagnosis and based on which biomarkers are needed when applicable.

| **NCT Number** | **Drug** | **Target class:** | **Phase** | **Duration (weeks)** | **N** | **Inclusion criteria** | **Biomarker endpoints** | **Clinical endpoints** | **pTau effect in report** |
| --- | --- | --- | --- | --- | --- | --- | --- | --- | --- |
| NCT04524351^1^ | Buntanetap | Amyloid β | Phase 2 | 4 | 75 | 45+; AD based on CDR-SB and MMSE scores | CSF Aβ40: NS compared to placebo  CSF Aβ42: NS compared to placebo  CSF tTau: NS compared to placebo  CSF pTau: NS compared to placebo  CSF sAPPa: NS compared to placebo  CSF sAPPb: NS compared to placebo  CSF sTREM2: NS compared to placebo  CSF GFAP: NS compared to placebo  CSF YKL-40: NS compared to placebo  CSF complement 3: NS compared to placebo  CSF NFL: NS compared to placebo  CSF NRGN: NS compared to placebo  Study not powered to measure statistically significant differences, trends were visible. | ADAS-Cog11: Better score compared to baseline  WAIS: Better score compared to baseline  MMSE: NS compared to baseline  CDR-SB: NS compared to baseline | NA |
| NCT03114657^2^ | Crenezumab | Amyloid β | Phase 3 | 100 | 805 | 50-85; MCI or probable dementia according to NIA-AA; Amyloid PET or CSF | Discontinued due to earlier study not meeting primary endpoint | Discontinued due to earlier study not meeting primary endpoint | NA |
| NCT04437511^3^ | Donanemab | Amyloid β | Phase 3 | 76 | 1800 | 60-85; gradual and progressive change in memory; Tau PET and amyloid PET | Plasma pTau217: decreased compared to placebo | iADRS: Better score compared to placebo | Plasma pTau217: Log_10_ -0.2 |
| NCT03367403^4,5^ | Donanemab | Amyloid β | Phase 2 | 72 | 266 | 60-85; gradual and progressive change in memory; positive Amyloid and Tau PET | Plasma pTau217: decreased compared to placebo  Plasma GFAP: decreased compared to placebo  Plasma NFL: NS compared to placebo  Plasma Aβ42/40: NS compared to placebo | iADRS: Better score compared to placebo  ADAS-Cog13: Inconclusive  CDR-SB/ADCS-iADL/MMSE: NS compared to placebo | Plasma pTau217: Log_10_ -0.14 |
| NCT03444870^6^ | Gantenerumab | Amyloid β | Phase 3 | 116 | 1016 | 50-90; NIA-AA for probable AD dementia or prodromal AD; AD confirmed by CSF tau/Aβ42 or amyloid PET scan | CSF tTau: decreased compared to placebo  CSF pTau181: decreased compared to placebo  CSF Aβ40: decreased compared to placebo  CSF Aβ42: increased compared to placebo  CSF NRGN: decreased compared to placebo  CSF NFL: decreased compared to placebo  Plasma pTau181: decreased compared to placebo  Plasma Aβ42: Increased compared to placebo | CDR-SB: NS compared to placebo  ADAS-Cog13: NS compared to placebo  ADCS-ADL: NS compared to placebo | CSF pTau181:  -23.8%  Plasma pTau181:  -24% |
| NCT03443973^6^ | Gantenerumab | Amyloid β | Phase 3 | 116 | 982 | 50-90; NIA-AA for probable AD dementia or prodromal AD; AD confirmed by CSF tau/Aβ42 or amyloid PET scan | CSF tTau: decreased compared to placebo  CSF pTau181: decreased compared to placebo  CSF Aβ40: decreased compared to placebo  CSF Aβ42: increased compared to placebo  CSF NRGN: decreased compared to placebo  CSF NFL: decreased compared to placebo  Plasma pTau181: decreased compared to placebo  Plasma Aβ42: increased compared to placebo | CDR-SB: NS compared to placebo  ADAS-Cog13: NS compared to placebo  ADCS-ADL: NS compared to placebo | CSF pTau181:  -23.8%  Plasma pTau181:  -21% |
| NCT03887455^7^ | Lecanemab | Amyloid β | Phase 3 | 78 | 1766 | 50-90; criteria of NIA-AA for MCI of probable AD; positive biomarker amyloid | CSF Aβ42: increased compared to placebo  CSF tTau: decreased compared to placebo  CSF pTau181: decreased compared to placebo  CSF NRGN: decreased compared to placebo  CSF Aβ40: NS compared to placebo  CSF NFL: NS compared to placebo  Plasma Aβ42/40: increased compared to placebo  Plasma pTau181: decreased compared to placebo  Plasma GFAP: decreased compared to placebo  Plasma NFL: decreased compared to placebo | CDR-SB: Better score compared to placebo  ADAS-Co14: Better score compared to placebo  ADCOMS: Better score compared to placebo  ADCS_MCI-ADL: Better score compared to placebo | CSF pTau181: ~30 pg/mL compared to placebo  -16 pg/mL compared to baseline  Plasma pTau181: ~0.8 pg/mL |
| NCT03706885^8^ | Efavirenz | ApoE, Lipids and Lipoprotein Receptors | Phase 1 | 52 | 5 | 55-85; AD confirmed by cognitive decline, MMSE 16-30 and CDR | Plasma 24-OHC: Increased compared to baseline  CSF Aβ40: NS compared to baseline  CSF Aβ42: NS compared to baseline  CSF tTau: NS compared to baseline  CSF pTau181: NS compared to baseline | MoCA: NS compared to baseline | NA |
| NCT03101085^9^ | S-equol | growth factors and hormones | Phase 2 | 4 | 40 | 50-90; AD diagnosis; exclude ApoE4 | COX/CS: increased compared to baseline | MoCA: NS compared to baseline | NA |
| NCT03757325^10^ | DNL747 | Inflammation | Phase 1 | 12 | 16 | 55-85; AD diagnosis based on NIA-AA, CSF Ab42 or Amyloid PET | Plasma PBMC pRIPK1: decreased compared to placebo | No clinical endpoints included | NA |
| NCT03402659^11^ | Neflamapimod | Inflammation | Phase 2 | 24 | 161 | 55-85; MCI or AD defined by CDR 0.5-1.0, MMSE 20-28; CSF Ab1-42, p-Tau, CT or MRI compatible with AD | CSF tTau: decreased compared to placebo  CSF pTau181: decreased compared to placebo  CSF NRGN: NS compared to placebo  CSF NFL: NS compared to placebo  CSF Aβ40: NS compared to placebo  CSF Aβ42: NS compared to placebo | HVLT-R/ WMS immediate and delayed recall/CDR-SB/ MMSE: NS compared to placebo | CSF pTau181:  -2.1 pg/mL |
| NCT04552795^12^ | 3TC (lamivudine) | Other | Phase 2 | 24 | 12 | 50-80; clinical diagnosis of early AD | CSF GFAP: decreased compared to baseline  Plasma Aβ42/40: increased compared to baseline  CSF NFL: NS compared to baseline  CSF Aβ42/40: NS compared to baseline  CSF pTau181: NS compared to baseline  Plasma NFL: NS compared to baseline  Plasma GFAP: NS compared to baseline  Plasma pTau181: NS compared to baseline | MMSE: NS compared to baseline  PACC-5: NS compared to baseline  Attention, memory, naming, and EF tasks: NS compared to baseline | NA |
| NCT03522129^13^ | CT1812 | Synaptic plasticity/ neuroprotection | Phase 1 | 1 | 3 | 50-80; mild to moderate AD based on NIA-AA; MRI and Abeta PET scan | CSF Aβ oligomers: Increased compared to baseline | No clinical endpoints included | NA |
| NCT03493282^14^ | CT1812 | Synaptic plasticity/ neuroprotection | Phase 2 | 30 | 23 | 50-85; Mild to moderate AD acoording to NIA-AA; Amyloid PET or Amyloid CSF | CSF Aβ40: NS compared to placebo  CSF Aβ42: NS compared to placebo  CSF tTau: NS compared to placebo  CSF pTau: NS compared to placebo  CSF NRGN: NS compared to placebo  CSF synaptotagmin: NS compared to placebo  CSF SNAP25: NS compared to placebo  CSF NFL: NS compared to placebo | ADCS-ADL: High dose better scores compared to placebo  ADAS-Cog11: NS compared to placebo  MMSE: NS compared to placebo | NA |
| NCT03186989^15^ | BIIB080 (MAPT_rx_) | Tau | Phase 2 | 61 | 46 | 50-74; mild Alzheimer's Disease diagnosis; CSF biomarkers | CSF tTau: decreased compared to placebo  CSF pTau181: decreased compared to placebo  CSF tTau/Aβ42: decreased compared to placebo  CSF NFL: NS compared to baseline  CSF NFH: NS compared to baseline  CSF NRGN: NS compared to baseline  CSF YKL-40: NS compared to baseline | RBANS Total score: NS compared to baseline  MMSE Total score: NS compared to baseline  NPI-Q/FAQ Total score: NS compared to baseline | CSF pTau181: Ranging from 0 to  ~-55% based on dose |
| NCT03352557^16^ | Gosuranemab | Tau | Phase 2 | 238 | 654 | 50-80; MCI or mild AD; positive for amyloid beta | CSF Unbound N-terminal tau: decreased in treatment compared to placebo  CSF pTau181: Decreased in high dose treatment compared to placebo  CSF tTau: Decreased in treatment compared to placebo  CSF Aβ42: NS compared to placebo | CDR-SB/MMSE/ADCS-ADL/FAQ: NS compared to placebo group  ADAS-Cog13: Significantly worse in treatment compared to placebo | CSF pTau181:  ~-25 pg/mL compared to placebo  -7.1 pg/mL compared to baseline |
| NCT03289143^17^ | Semorinemab | Tau | Phase 2 | 73 | 457 | 50-80; probable AD or MCI according to NIA-AA; Amyloid PET or CSF | Plasma mid-domain tTau: increased compared to placebo  CSF tTau: decreased from baseline  CSF pTau181: decreased from baseline | CDR-SB/ADAS-Cog13/RBANS/  ADCS-ADL/A-IADL-Q: NS compared to placebo | CSF pTau181:  -9.7 pg/mL compared to placebo  -10.5 pg/mL compared to baseline |
| NCT03828747^18^ | Semorinemab | Tau | Phase 2 | 72 | 273 | 50-85; probable AD according to NIA-AA, MMSE 16-21; CSF Ab42 or Amyloid PET | Plasma tTau: increased compared to placebo  Plasma pTau217: increased compared to placebo  CSFpTau217: decreased compared to placebo  CSF pTau181: decreased compared to placebo  CSF tTau: decreased compared to placebo  CSF N-term Tau: NS compared to placebo | ADAS-Cog11: Better score compared to placebo  ADCS-ADL/CDR-SB/MMSE: NS compared to placebo | Plasma pTau217:  ~+88 pg/mL  CSF pTau217:  ~-50%  CSF pTau181:  ~-12% |
| NCT03518073^19^ | Zagotenemab | Tau | Phase 2 | 104 | 360 | 60-85; AD, progressive change in memory > 6m | Plasma tTau: Increased compared to placebo  Plasma pTau181: Increased compared to placebo  Plasma NFL: NS compared to placebo | iADRS/ADCS-iADL/ADAS-Cog13/CDR-SB/MMSE: NS compared to placebo | Plasma pTau181: ~+15 pg/mL (low dose);  ~+ 30 pg/mL (high dose) |
| NCT03019536^20^ | Zagotenemab | Tau | Phase 1 | 64 | 24 | 50 +; MCI-AD /mild/moderate AD based on NIA-AA | Plasma tTau: NS compared to placebo | No clinical endpoints included | NA |

1 Fang, C. *et al.* Buntanetap, a Novel Translational Inhibitor of Multiple Neurotoxic Proteins, Proves to Be Safe and Promising in Both Alzheimer's and Parkinson's Patients. *J Prev Alzheimers Dis* **10**, 25-33 (2023). <https://doi.org:10.14283/jpad.2022.84>

2 Ostrowitzki, S. *et al.* Evaluating the Safety and Efficacy of Crenezumab vs Placebo in Adults With Early Alzheimer Disease: Two Phase 3 Randomized Placebo-Controlled Trials. *JAMA Neurology* **79**, 1113-1121 (2022). <https://doi.org:10.1001/jamaneurol.2022.2909>

3 Sims, J. R. *et al.* Donanemab in Early Symptomatic Alzheimer Disease: The TRAILBLAZER-ALZ 2 Randomized Clinical Trial. *JAMA* **330**, 512-527 (2023). <https://doi.org:10.1001/jama.2023.13239>

4 Mintun, M. A. *et al.* Donanemab in Early Alzheimer’s Disease. *New England Journal of Medicine* **384**, 1691-1704 (2021). <https://doi.org:10.1056/NEJMoa2100708>

5 Pontecorvo, M. J. *et al.* Association of Donanemab Treatment With Exploratory Plasma Biomarkers in Early Symptomatic Alzheimer Disease: A Secondary Analysis of the TRAILBLAZER-ALZ Randomized Clinical Trial. *JAMA Neurology* (2022). <https://doi.org:10.1001/jamaneurol.2022.3392>

6 Bateman, R. J. *et al.* Two Phase 3 Trials of Gantenerumab in Early Alzheimer’s Disease. *New England Journal of Medicine* **389**, 1862-1876 (2023). <https://doi.org:10.1056/NEJMoa2304430>

7 van Dyck, C. H. *et al.* Lecanemab in Early Alzheimer’s Disease. *New England Journal of Medicine* (2022). <https://doi.org:10.1056/NEJMoa2212948>

8 Lerner, A. J. *et al.* CYP46A1 activation by low-dose efavirenz enhances brain cholesterol metabolism in subjects with early Alzheimer's disease. *Alzheimers Res Ther* **14**, 198 (2022). <https://doi.org:10.1186/s13195-022-01151-z>

9 Wilkins, H. M. *et al.* A Mitochondrial Biomarker-Based Study of S-Equol in Alzheimer's Disease Subjects: Results of a Single-Arm, Pilot Trial. *J Alzheimers Dis* **59**, 291-300 (2017). <https://doi.org:10.3233/jad-170077>

10 Vissers, M. F. J. M. *et al.* Safety, pharmacokinetics and target engagement of novel RIPK1 inhibitor SAR443060 (DNL747) for neurodegenerative disorders: Randomized, placebo-controlled, double-blind phase I/Ib studies in healthy subjects and patients. *Clinical and Translational Science* **15**, 2010-2023 (2022). <https://doi.org:https://doi.org/10.1111/cts.13317>

11 Prins, N. D. *et al.* A phase 2 double-blind placebo-controlled 24-week treatment clinical study of the p38 alpha kinase inhibitor neflamapimod in mild Alzheimer’s disease. *Alzheimer's Research & Therapy* **13**, 106 (2021). <https://doi.org:10.1186/s13195-021-00843-2>

12 Sullivan, A. C. *et al.* A pilot study to investigate the safety and feasibility of antiretroviral therapy for Alzheimer's disease (ART-AD). *medRxiv* (2024). <https://doi.org:10.1101/2024.02.26.24303316>

13 LaBarbera, K. M. *et al.* A phase 1b randomized clinical trial of CT1812 to measure Aβ oligomer displacement in Alzheimer's disease using an indwelling CSF catheter. *Transl Neurodegener* **12**, 24 (2023). <https://doi.org:10.1186/s40035-023-00358-w>

14 van Dyck, C. H. *et al.* A pilot study to evaluate the effect of CT1812 treatment on synaptic density and other biomarkers in Alzheimer’s disease. *Alzheimer's Research & Therapy* **16**, 20 (2024). <https://doi.org:10.1186/s13195-024-01382-2>

15 Mummery, C. J. *et al.* Tau-targeting antisense oligonucleotide MAPTRx in mild Alzheimer’s disease: a phase 1b, randomized, placebo-controlled trial. *Nature Medicine* **29**, 1437-1447 (2023). <https://doi.org:10.1038/s41591-023-02326-3>

16 Shulman, M. *et al.* TANGO: a placebo-controlled randomized phase 2 study of efficacy and safety of the anti-tau monoclonal antibody gosuranemab in early Alzheimer’s disease. *Nature Aging* **3**, 1591-1601 (2023). <https://doi.org:10.1038/s43587-023-00523-w>

17 Teng, E. *et al.* Safety and Efficacy of Semorinemab in Individuals With Prodromal to Mild Alzheimer Disease: A Randomized Clinical Trial. *JAMA Neurology* **79**, 758-767 (2022). <https://doi.org:10.1001/jamaneurol.2022.1375>

18 Monteiro, C. *et al.* Randomized Phase II Study of the Safety and Efficacy of Semorinemab in Participants With Mild-to-Moderate Alzheimer Disease: Lauriet. *Neurology* **101**, e1391-e1401 (2023). <https://doi.org:10.1212/wnl.0000000000207663>

19 Fleisher, A. S. *et al.* Assessment of Efficacy and Safety of Zagotenemab. *Neurology* **102**, e208061 (2024). <https://doi.org:10.1212/WNL.0000000000208061>

20 Willis, B. A. *et al.* Safety, Tolerability, and Pharmacokinetics of Zagotenemab in Participants with Symptomatic Alzheimer's Disease: A Phase I Clinical Trial. *J Alzheimers Dis Rep* **7**, 1015-1024 (2023). <https://doi.org:10.3233/adr-230012>
